# *Letter to the Editor:* A Favorable Benefit–Risk Balance Maybe Expected with Replication-Defective Adenovirus-Mediated Interferon Gene Therapy for Cancer Treatment

**DOI:** 10.1089/hum.2023.028

**Published:** 2023-04-17

**Authors:** Albert Qin

**Affiliations:** Medical Research and Clinical Operations, PharmaEssentia Corporation, Taipei, Taiwan.

**To the Editor:**

The United States Food and Drug Administration approved a nonreplicating adenovirus encoding interferon alfa-2b (IFN-α2b) for high-risk Bacillus Calmette-Guérin (BCG)-unresponsive non-muscle invasive bladder cancer on December 16, 2022,^1^ paving the way for further approval of gene therapy treatment for cancer. Adenoviral vectors can be easily produced in large quantities and deliver high gene expression levels to elicit antitumor effect. IFN-α and IFN-β can bind to the same receptor component to induce similar antitumor activities.

A replication-defective adenovirus-mediated IFN gene therapy developed more than 25 years ago provided significant insights into this gene therapy approach for cancer treatment.^2^ The idea was to use a replication-defective adenoviral vector to overexpress IFN-β to suppress tumors without an intrapatient dissemination risk from using a replication-competent virus. Direct intratumor gene therapy with a replication-defective IFN-β-encoding adenovirus led to a remarkable suppression of human xenograft tumors in mice, and the complete tumor disappearance.^2^

*Ex vivo* gene therapy by mixing the virus-infected tumor cells with uninfected tumor cells further revealed the substantial antitumor effect. Mixing 1% virus-infected tumor cells with 99% uninfected tumor cells blocked tumorigenicity *in vivo*. Mixing only 0.1% infected cells also significantly suppressed tumor formation, indicating that infecting very few cells, such as 1 out of 1000, by this gene therapy could be adequate in suppressing tumors by a potent bystander effect.^2^ Subsequent animal modeling showed that a replication-defective adenovirus-mediated IFN-β gene therapy could also activate immunity-mediated antitumor effect.^3^

Direct evidence that IFN-β functions as a tumor suppressor protein, as shown with a lentiviral transduction,^4^ also supports the utility of this regimen for cancer treatment. Our findings validate that replication-defective adenovirus-mediated IFN therapy is an effective antitumor treatment and suggest that very few cancer cells inside a tumor need to be infected for achieving a significant antitumor effect.^2–4^ The virus leads to notable apoptosis inside tumors,^2^ which can clear out the directly infected cells. Therefore, a favorable benefit–risk balance in clinical use is expected as this approach can lead to strong antitumor effects and the risk of side effects from the continued presence of the virus and IFN expression can be minimal.

This does not exclude a possibility that an oncolytic virus carrying an IFN is also effective with manageable toxicities. However, the gene therapy animal model data with a replication-defective adenovirus encoding IFN and its clinical trial validation indicate that the replication-defective viral approach to overexpress IFN is a reasonable treatment option for certain human tumors.

Our recent data on ropeginterferon alfa-2b, a new-generation pegylated IFN, indicate that patients can tolerate its higher doses, suggesting that certain levels of IFN in the circulation system do not cause an unmanageable safety risk.^5^ With the technical advances in the image-guided clinical deliveries and gene therapy vector production, it is conceivable that a replication-defective human IFN-α or -β-encoding adenovirus can deliver promising treatment options to more cancer patients and could be applied in more cancer indications beyond the high-risk BCG-unresponsive non-muscle invasive human bladder cancer.
